# Contextual factors influencing implementation of tuberculosis digital adherence technologies: a scoping review guided by the RE-AIM framework

**DOI:** 10.1136/bmjgh-2024-016608

**Published:** 2025-02-13

**Authors:** Shruti Bahukudumbi, Chimweta I Chilala, Nicola Foster, Barbie Patel, Mona S Mohamed, Miranda Zary, Cedric Kafie, Genevieve Gore, Kevin Schwartzman, Katherine L Fielding, Ramnath Subbaraman

**Affiliations:** 1 Department of Public Health and Community Medicine and Center for Global Public Health, Tufts University School of Medicine, Boston, Massachusetts, USA; 2 TB Centre, London School of Hygiene and Tropical Medicine, London, UK; 3 McGill International Tuberculosis Centre, Research Institute of the McGill University Health Centre, Montreal, Quebec, Canada; 4 Schulich Library of Physical Sciences, Life Sciences, and Engineering, McGill University, Montreal, Quebec, Canada; 5 Division of Geographic Medicine and Infectious Diseases, Tufts Medical Center, Boston, Massachusetts, USA

**Keywords:** Tuberculosis, Global Health, Systematic review

## Abstract

**Introduction:**

Digital adherence technologies (DATs) may enable person-centred tuberculosis (TB) treatment monitoring; however, implementation challenges may undermine their effectiveness. Using the reach, effectiveness, adoption, implementation and maintenance framework, we conducted a scoping review to identify contextual factors informing ‘reach’ (DAT engagement by people with TB) and ‘adoption’ (DAT uptake by healthcare providers or clinics).

**Methods:**

We searched eight databases from 1 January 2000 to 25 April 2023 to identify all TB DAT studies. After extracting qualitative and quantitative findings, using thematic synthesis, we analysed common findings to create meta-themes informing DAT reach or adoption. Meta-themes were further organised using the Unified Theory of Acceptance and Use of Technology, which posits technology use is influenced by perceived usefulness, ease of use, social influences and facilitating conditions.

**Results:**

66 reports met inclusion criteria, with 61 reporting on DAT reach among people with TB and 27 reporting on DAT adoption by healthcare providers. Meta-themes promoting reach included perceptions that DATs improved medication adherence, facilitated communication with providers, made people feel more ‘cared for’ and enhanced convenience compared with alternative care models (perceived usefulness) and lowered stigma (social influences). Meta-themes limiting reach included literacy and language barriers and DAT technical complexity (ease of use); increased stigma (social influences) and suboptimal DAT function and complex cellular accessibility challenges (facilitating conditions). Meta-themes promoting adoption included perceptions that DATs improved care quality or efficiency (perceived usefulness). Meta-themes limiting adoption included negative DAT impacts on workload or employment and suboptimal accuracy of adherence data (perceived usefulness); and suboptimal DAT function, complex cellular accessibility challenges and insufficient provider training (facilitating conditions). Limitations of this review include the limited studies informing adoption meta-themes.

**Conclusion:**

This review identifies diverse contextual factors that can inform improvements in DAT design and implementation to achieve higher engagement by people with TB and healthcare providers, which could improve intervention effectiveness.

**WHAT IS ALREADY KNOWN ON THIS TOPIC**

- Digital adherence technologies (DATs) are increasingly used to monitor tuberculosis (TB) treatment; however, systematic reviews suggest DATs have mixed effectiveness for improving TB outcomes and suboptimal accuracy for measuring medication adherence.
- Inadequate DAT ‘reach’ (engagement by people with TB) and ‘adoption’ (uptake by healthcare providers) may contribute to their limited effectiveness and accuracy.
- Understanding contextual factors influencing DAT reach and adoption may be critical to improve the design, implementation and public health impact of TB DATs.

**WHAT THIS STUDY ADDS**

**HOW THIS STUDY MIGHT AFFECT RESEARCH, PRACTICE OR POLICY**

- Our findings may inform future design of DATs to focus on what people with TB value, such as improved communication with providers and convenience of care.
- Our findings may also help to identify settings in which DATs are unlikely to be effective, such as locations where cellular accessibility barriers are substantial due to poor infrastructure.

## Introduction

 Digital adherence technologies (DATs) have been increasingly studied and implemented as part of routine tuberculosis (TB) care over the last decade.^1 2^ These technologies—which include interventions involving short messaging service (SMS) texting, feature (non-smart) phones, video-supported therapy (VST), digital pillboxes, and ingestion sensors—provide reminders and enable electronic observation of dose ingestion, often with the aim of replacing in-person direct observation of therapy (DOT).

Despite this enthusiasm, evaluations of the evidence regarding TB DATs reveal challenges limiting their public health impact.^3 4^ For example, a recent systematic review evaluating the effectiveness of DATs for improving TB treatment outcomes revealed mixed findings.^5^ Although some DATs, like VST, were associated with improved treatment outcomes in high-income and upper-middle-income countries, most technologies evaluated in low-income or lower-middle-income countries, which account for most people with TB globally, were not associated with improved outcomes. Another recent systematic review evaluated the performance of DATs for measuring TB medication adherence because an important function of some DATs is the identification of non-adherent individuals who may need additional support.^6^ This review found DATs often have variable or suboptimal performance for measuring adherence in real-world conditions.

The limited effectiveness and accuracy of TB DATs may be explained, in part, by implementation challenges. RE-AIM (reach, effectiveness, adoption, implementation and maintenance) is a framework for assessing implementation outcomes and contextual factors.^7^ Two RE-AIM outcomes are of particular relevance to this paper. ‘Reach’ refers to the proportion of people who receive an intended public health intervention; we use reach to refer to the proportion of people taking TB treatment who receive or engage with DAT interventions. ‘Adoption’ refers to the proportion of healthcare providers or settings that engage in delivering a public health intervention; we use adoption to refer to the proportion of healthcare providers or clinics appropriately delivering or engaging with DAT interventions.

A forthcoming scoping review evaluating DAT implementation outcomes shows that serial drop-offs in DAT engagement by people with TB—for example, suboptimal cellphone access, provision of DATs to people with TB or initial or sustained engagement by people with TB—contribute to poor overall reach of DATs in many settings, especially low-income and lower-middle-income countries.^8^ Although findings on adoption were more limited, the review also found high variability in DAT adoption across clinics in some settings. While that review quantified implementation outcomes, its findings did not attempt to provide insights into contextual barriers contributing to suboptimal reach and adoption.

In this paper, which is a companion to the DAT implementation outcomes scoping review, we report findings of a scoping review aimed at identifying contextual factors informing the reach and adoption of DATs. We conducted a scoping review, rather than a systematic review, given the heterogeneity of study types reporting contextual findings and the flexibility that a scoping review allows in combining different findings using thematic synthesis.^9^ We further organised findings using constructs in the Unified Theory of Acceptance and Use of Technology (UTAUT), which helps to understand and predict technology engagement.^10^ By understanding contextual barriers, this review may inform the design and implementation of future DAT interventions so as to enhance their reach, adoption, effectiveness and public health impact.

## Methods

### Population, Intervention, Comparison, Outcomes and Study framework and scoping review design

The review was conceived as a systematic review and registered in PROSPERO, the International Prospective Register of Systematic Reviews (CRD42022326968). During data extraction, based on the heterogeneity in study designs, we modified this to a scoping review, though we followed the original protocol except that we did not assess study quality or risk of bias. We followed the Preferred Reporting Items for Systematic Reviews and Meta-Analyses Extension for Scoping Reviews guideline (checklist is in online supplemental appendix 1).^11^

Our study design is guided by a Population, Intervention, Comparison, Outcomes and Study design framework.^12^ For the RE-AIM reach outcome, the population includes people being treated for TB disease or infection, including children with TB, people with drug-resistant TB and people with HIV. For the RE-AIM adoption outcome, the population includes healthcare providers, programme managers or policymakers involved in DAT implementation. The intervention comprises TB DATs, defined as an intervention with a digital component (which could be part of a multicomponent intervention) intending to measure or promote adherence, reduce missed clinic visits or reduce loss to follow-up. Studies may or may not have included a comparison group. When reported, comparison groups usually comprised various DOT approaches (eg, clinic-based DOT, family DOT) or self-administered therapy. Outcomes included all RE-AIM dimensions; however, we only report findings on DAT reach and adoption, given limited contextual findings on other RE-AIM dimensions.^7^ We extracted qualitative contextual findings (eg, emergent themes from interviews or focus groups) and quantitative contextual findings from structured surveys (eg, percentage of people reporting specific benefits of DATs).

### Search strategy

Initial and refresher searches without language restrictions were conducted by a librarian, collectively spanning 1 January 2000 to 25 April 2023 (updated from 14 April 2022), in MEDLINE/Ovid, Embase, the Cochrane Central Register of Controlled Trials, CINAHL, Web of Science, ClinicalTrials.gov and Europe PMC to capture preprints (including those in medRxiv). As any TB DAT study could report contextual findings on implementation, our search strategy was broad, including only two concepts with related terms: “tuberculosis” (including TB disease and infection) and “digital adherence technologies,” including terms for specific TB DATs (the full search strategy is in online supplemental appendix 1). Finally, we extracted and screened all references from systematic reviews identified in our initial search to assess the validity of our search and identify outstanding studies. We found no further studies meeting the inclusion criteria. Although our search did not involve language restrictions, all studies identified for inclusion were in the English language.

### Inclusion and exclusion criteria

We included studies if they reported on DAT implementation (not hypothetical use) during treatment for TB disease or infection and if they reported contextual findings on at least one RE-AIM outcome, excluding effectiveness. We excluded effectiveness as an outcome in this review, because effectiveness is usually measured quantitatively, and these findings are the subject of a separate systematic review.^5^ In addition, because contextual factors informing reach and adoption capture perspectives from people with TB and healthcare providers regarding their engagement with DATs, these factors are also likely to influence whether DATs will be effective for improving TB outcomes. Although data collection may have been embedded within other study designs (eg, cohort studies or randomised trials), relevant contextual findings usually involved qualitative interviews, structured surveys or direct observation of people with TB or healthcare providers. After extracting data, sufficient contextual findings were only available for RE-AIM outcomes of reach and adoption, though the RE-AIM implementation outcome (ie, the fidelity with which providers adhere to an intervention protocol) is indirectly informed by our review findings as described later.

We excluded protocols, review articles, editorials and commentaries; articles in which the technology was not used to measure or improve adherence; and articles in which clinical or implementation outcomes were reported without contextual findings.

### Screening strategy and study selection

After deduplication using EndNote (V.20.2.1, Clarivate, London, UK), two reviewers (among SB, CIC, MSM, MZ, CK and NF) independently screened titles and abstracts in a blinded manner using Rayyan.ai (Cambridge, USA). Full-text articles were independently assessed in a blinded manner for inclusion by two reviewers, with conflicts resolved by senior investigators (RS, KLF or KS).

### Data extraction

Data from the included studies were extracted into an Excel template by five reviewers (SB, CIC, NF, KLF and RS). Given the large number of articles, for each article, one reviewer extracted data and a second verified these data, with conflicts resolved through discussion or involvement of a third reviewer. We extracted data on study characteristics, design, setting (inpatient or outpatient), participant characteristics, type of DAT and details on the intervention programme in which the DAT was embedded. We extracted contextual findings into separate Excel sheets for RE-AIM outcomes of reach, adoption, implementation and maintenance, though findings on the latter two outcomes were extremely limited. We separated qualitative and quantitative contextual findings and included separate columns for each UTAUT construct (described further below).

### Data analysis: synthesis of meta-themes and reporting using the UTAUT

After completing data extraction, authors SB and RS engaged in an iterative process using thematic synthesis to create new ‘meta-themes’ that may inform increased or suboptimal reach or adoption. Thematic synthesis is an extension of meta-ethnography originally described by Noblit and Hare for conducting qualitative systematic reviews,^9 13 14^ an approach that has previously been used for TB qualitative systematic reviews.^15^ Our approach to thematic synthesis involved aggregating similar themes into a new meta-theme that reflects a broader concept from the underlying findings. More specifically, author SB copied extracted findings onto a new Excel spreadsheet, which organised findings by whether they informed increased or suboptimal reach. Within increased or suboptimal reach, new sheets were created to organise themes by each UTAUT construct. SB and RS met on a weekly basis over a few months to iteratively group similar themes together and then create meta-themes to reflect broader underlying concepts, with routine feedback from CIC, NF and KLF. The same process was followed for adoption.

Our thematic synthesis approach is unique in that we included both qualitative and quantitative data as contextual findings, as there was similarity in concepts across both types of data. For a quantitative finding to be included in the synthesis, it had to be reported by at least 15% of people surveyed to indicate that it had a meaningful influence on reach or adoption. For adverse effects of DATs or concerns related to stigma, privacy or confidentiality only, we included quantitative findings if reported by at least 5% of people surveyed, as such challenges could be associated with significant harm (eg, disclosure of TB diagnosis). Given our scoping review approach, we did not evaluate or exclude studies based on quality, and this is actually consistent with the approach taken by many qualitative systematic reviews.^15^

Final meta-themes were reported using the UTAUT, which describes the following constructs that influence technology use: performance expectancy, effort expectancy, social influences and facilitating conditions.^10^ Performance expectancy, or perceived usefulness, is the extent to which a person thinks the DAT helps with TB care (for people with TB) or care delivery (for healthcare providers). Effort expectancy, or ease of use, refers to how easily people are able to navigate and use the DAT. Social influences refer to the role of other individuals (eg, family members) or societal factors in influencing DAT use. Facilitating conditions refer to the quality of institutional support or infrastructure to ensure success of the DAT intervention. Throughout this paper, we use ‘perceived usefulness’ and ‘ease of use’ to refer to performance expectancy and effort expectancy, respectively, given the more intuitive meaning of the former terms.

### Patients and public involvement

Patients or the public were not involved in the design, or conduct, or reporting, or dissemination plans of our research.

## Results

### Characteristics of the included studies

**Figure 1:**
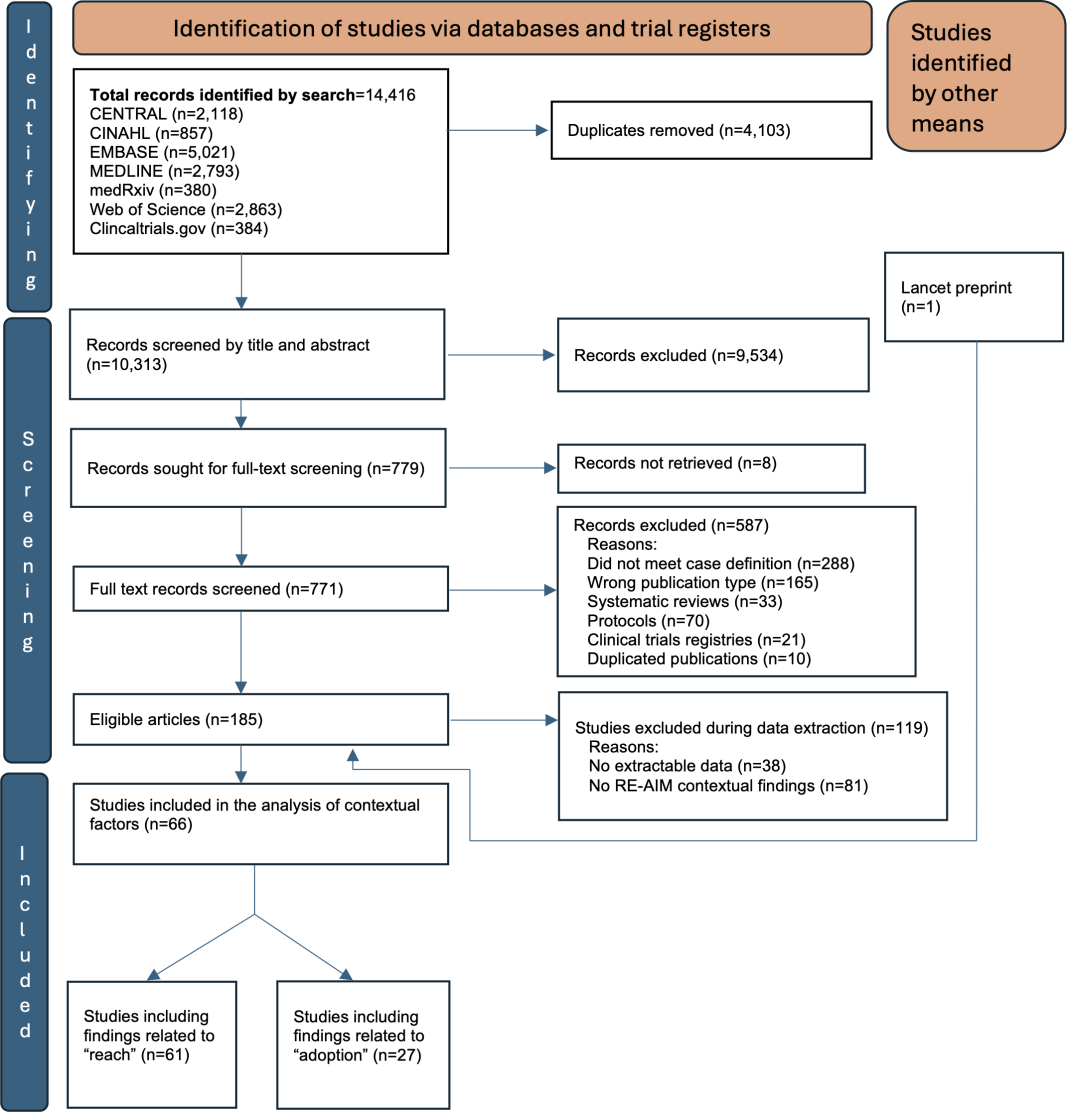
PRISMA flow diagram summarising the process of identifying included articles. PRISMA, Preferred Reporting Items for Systematic Reviews and Meta-Analyses; RE-AIM, reach, effectiveness, adoption, implementation and maintenance.

Of 14 416 abstracts identified through the search, 771 underwent full-text review, of which 66 had relevant data for extraction (table 1 and figure 1). Of the included articles, 61/66 (92%) reported contextual findings on reach of DATs among people with TB, while 27/66 (41%) reported on DAT adoption by healthcare providers. Of the included articles, the primary DAT was SMS in 14 studies, feature (non-smart) phone-based approaches (including 99DOTS) in 10 studies, VST in 24 studies, digital pillboxes in 12 studies, ingestion sensors in 2 studies and App-based in 5 studies. 99DOTS is a feature phone-based DAT requiring people with TB to call a number daily to report medication ingestion.^16^

**Table 1:** Characteristics of studies included in this scoping review evaluating contextual factors influencing implementation of TB digital adherence technologies

Author year	Dominant DAT modality	Type of TB	Countries	Country income level	RE-AIM dimension (ie, reach, adoption, or both)	Type of data reported (ie, qualitative, quantitative or both)
Bardosh 2017^17^	Phone	TB Infection	Canada, Kenya	High, lower middle	Both	Both
Bassett 2016^68^	SMS	Drug-sensitive TB	South Africa	Upper middle	Reach	Quantitative
Bediang 2018^86^	SMS	Drug-sensitive TB	Cameroon	Lower middle	Reach	Quantitative
Belknap 2013^87^	Ingestion Sensor	Drug-sensitive TB	USA	High	Reach	Quantitative
Bendiksen 2020^29^	VST	Drug-sensitive TB	Norway	High	Both	Both
Bionghi 2018^47^	Digital Pillbox	Drug-resistant TB	South Africa	Upper middle	Reach	Both
Bommakanti 2020^54^	VST	Drug-sensitive TB	USA	High	Reach	Quantitative
Browne 2019^30^	Ingestion Sensor	Drug-sensitive TB	USA	High	Reach	Quantitative
Buchman 2017^55^	VST	Drug-sensitive TB	USA	High	Reach	Qualitative
Burzynski 2022^46^	VST	Drug-sensitive TB	USA	High	Reach	Quantitative
Chen 2020^31^	VST	TB Infection	Taiwan, China	High	Reach	Quantitative
Chuck 2016^60^	VST	Drug-sensitive TB	USA	High	Reach	Quantitative
Cox 2018^58^	SMS	Drug-sensitive TB	India	Lower middle	Reach	Quantitative
Cross 2019^16^	99DOTS	Drug-sensitive TB	India	Lower middle	Reach	Qualitative
Daftary 2017^18^	Phone	TB Infection	Ethiopia	Low	Both	Both
Das Gupta 2020^50^	Phone	Drug-sensitive TB	India	Lower middle	Reach	Qualitative
de Sumari-de Boer 2016^61^	Digital Pillbox	Drug-sensitive TB	Tanzania	Lower middle	Reach	Qualitative
DeMaio 2001^32^	VST	Drug-sensitive TB	USA	High	Reach	Quantitative
Dessie Gashu 2020^51^	Phone	Drug-sensitive TB	Ethiopia	Low	Reach	Both
Drabarek 2019^63^	Digital Pillbox	Drug-sensitive TB	Vietnam	Lower middle	Both	Qualitative
Garfein 2020^48^	VST	Drug-sensitive TB	USA	High	Reach	Quantitative
Garfein 2015^33^	VST	Drug-sensitive TB	USA, Mexico	High, upper middle	Reach	Quantitative
Gashu 2021^66^	SMS	Drug-sensitive TB	Ethiopia	Low	Reach	Qualitative
Gassanov 2013^34^	VST	Drug-sensitive TB	Canada	High	Reach	Qualitative
Getachew 2022^71^	Digital Pillbox	Drug-sensitive TB	Ethiopia	Low	Adoption	Both
Guo 2020^35^	VST	Drug-sensitive TB	China	Upper middle	Reach	Quantitative
Guo 2020^36^	VST	Drug-sensitive TB	China	Upper middle	Reach	Quantitative
Hermans 2017^49^	SMS	Drug-sensitive TB	Uganda	Low	Reach	Both
Hirsch-Moverman 2017^19^	SMS	Drug-sensitive TB	South Africa	Upper middle	Both	Both
Hoffman 2010^37^	VST	Drug-sensitive TB	Kenya	Lower middle	Both	Both
Holzman 2018^38^	VST	Drug-sensitive TB	USA	High	Both	Both
Holzman 2019^62^	VST	Drug-sensitive TB	India	Lower middle	Reach	Quantitative
Horter 2014^88^	App-based	Drug-resistant TB	UK, Australia, Philippines, Swaziland, Central African Republic, Uganda, South Africa, India, Armenia	High, high, lower middle	Both	Qualitative
Iribarren 2020^20^	App-based	Drug-sensitive TB	Argentina	Upper middle	Reach	Qualitative
Iribarren 2015^21^	SMS	Drug-sensitive TB	Argentina	Upper middle	Both	Both
Iribarren 2013^22^	SMS	Drug-sensitive TB	Argentina	Upper middle	Both	Both
Kalita 2021^89^	Phone	Drug-sensitive TB	India	Lower middle	Reach	Both
Khachadourian 2020^90^	SMS	Drug-sensitive TB	Armenia	Upper middle	Reach	Quantitative
Kopanitsa 2017^59^	App-based	Drug-sensitive TB	Russian Federation	Upper middle	Both	Qualitative
Krueger 2010^72^	VST	Drug-sensitive TB	USA	High	Adoption	Quantitative
Lam 2018^65^	VST	TB Infection	USA	High	Both	Quantitative
Liu 2015^56^	SMS, Digital Pillbox	Drug-sensitive TB	China	Upper middle	Reach	Quantitative
Mahmud 2010^23^	SMS	Drug-sensitive TB	Malawi	Low	Both	Both
Milligan 2021^24^	App-based	Drug-sensitive TB	Argentina	Upper middle	Both	Qualitative
Mohammed 2016^67^	SMS	Drug-sensitive TB	Pakistan	Lower middle	Reach	Quantitative
Mohammed 2012^57^	SMS	Drug-sensitive TB	Pakistan	Lower middle	Reach	Both
Moulding 2002^91^	Digital Pillbox	Drug-sensitive TB	Haiti	Lower middle	Adoption	Quantitative
Navin 2017^25^	App-based	Drug-sensitive TB	India	Lower middle	Reach	Quantitative
Nhavoto 2017^26^	SMS	Drug-sensitive TB	Mozambique	Low	Reach	Both
Olano-Soler 2017^27^	VST	Drug-sensitive TB	USA	High	Both	Both
Person 2011^69^	Phone	TB Infection	USA	High	Reach	Qualitative
Prabhu 2021^52^	99DOTS	Drug-sensitive TB	India	Lower middle	Reach	Qualitative
Ratchakit-Nedsuwan 2020^28^	Digital Pillbox	Drug-sensitive TB	Thailand	Upper middle	Reach	Qualitative
Ritter 2017^73^	VST	Drug-sensitive TB	USA	High	Adoption	Qualitative
Sinkou 2017^39^	VST	Drug-sensitive TB, Drug-resistant TB	Belarus	Upper middle	Reach	Qualitative
Stagg 2020^92^	Digital Pillbox	Drug-sensitive TB	China	Upper middle	Reach	Qualitative
Story 2019^40^	VST	Drug-sensitive TB	UK	High	Both	Quantitative
Thekkur 2019^53^	99DOTS	Drug-sensitive TB	India	Lower middle	Both	Qualitative
Thomas 2021^41^	Digital Pillbox	Drug-resistant TB	India	Lower middle	Both	Qualitative
Thomas 2020^42^	99DOTS	Drug-sensitive TB	India	Lower middle	Both	Qualitative
Ting 2020^43^	VST	Drug-sensitive TB	Australia	High	Reach	Qualitative
Trajman 2010^64^	Digital Pillbox	TB Infection	Canada, Brazil, Saudi Arabia	High, Upper middle, High	Both	Qualitative
van den Boogaard 2011^93^	Digital Pillbox	Drug-sensitive TB	Tanzania	Lower middle	Reach	Quantitative
Wade 2012^44^	VST	Drug-sensitive TB	Australia	High	Both	Qualitative
Wade 2009^45^	VST	Drug-sensitive TB	Australia	High	Reach	Qualitative
Wang 2019^70^	Digital Pillbox	Drug-sensitive TB	China	Upper middle	Adoption	Quantitative

App, application; DAT, digital adherence technology; RE-AIM, reach, effectiveness, adoption, implementation, maintenance; SMS, short messaging service; TB, tuberculosis; VST, video-supported therapy

### Contextual factors promoting DAT reach among people with TB

With regard to the UTAUT construct of perceived usefulness, four meta-themes described perceptions that DATs enhanced people’s ability to engage in TB treatment: improved medication adherence behaviour, better storage and organisation of medications, enhanced access to information and knowledge, and better monitoring of medication adverse effects (table 2; findings for each reach meta-theme are in online supplemental file 2).

**Table 2:** Meta-themes *promoting* reach (ie, DAT use by people with TB) with findings organised using the UTAUT

Emerging meta-themes divided by UTAUT construct	Number of studies reporting meta-theme (citations)	DATs for which finding was reported (Number of studies per DAT)	Representative quotation on the meta-theme
Perceived usefulness			
Improved medication adherence behaviour	23 (1618 19 22 26 28 31 33 35,39 41 42 47 50 51 59 61 86 90 93)	SMS (6), Phone-based (4), 99DOTS (2), Digital pillbox (4), VST (6), App-based (1)	“(E)very time I forgot to take my medication, I was getting a reminder SMS then I find myself developing the habit of taking my pills…” (SMS)^61^“I have a new awareness that I should take tablets at 11 o’ clock. If we forget to call, the message alerts us…So it trains our mind to take pills on time…” (99DOTS)^42^
Better storage and organisation of medications	3 (41 47 61)	Digital pillbox (3)	“There are different compartments for each tablet, so they don’t get mixed…I like the arrows with the dots that explain how many of each medication I need to take.” (Digital pillbox)^41^
Enhanced access to information and knowledge	5 (20 22 24 26 37)	SMS (2), VST (1), App-based (2)	“…if you didn’t know where to look for information, this way you have it at hand [in application] and you do not have to go look for it in books…” (App-based)^20^
Better monitoring of medication adverse effects	5 (20 24 27 35 89)	Phone-based (1), VST (2), App-based (2)	Description of treatment supporter follow-up by App of adverse effects reported by a person with TB: “I saw that you reported nausea and vomiting. Is it a stomach ache? Tell me when you are taking your medication” (App-based)^24^
Facilitated communication with providers	12 (17,28)	SMS (5), Phone-based (2), Digital pillbox (1), VST (1), App-based (3)	“(I)t helps me to call and communicate with them when I am sick and receive calls from the clinic. Rather than totally depend on somebody, I can receive calls and I am able to call them when appropriate.’’ (Phone-based)^18^
Improved relationship between person with TB and provider	4 (24 41 42 44)	99DOTS (1), Digital pillbox (1), VST (1), App-based (1)	
Fostered a feeling of being ‘cared for’	11 (16,1922 41 42 47 57 66 93)	SMS (4), Phone-based (2), 99DOTS (2), Digital pillbox (3)	“If I don’t open the [pill] box, somebody from the health center calls me to find out whether I have taken the tablets or not. They care for me.” (Digital pillbox)^41^“It made me feel very important to have people worried about how I take my medication.” (Digital pillbox)^47^
Convenience of care delivery	5 (17 21 38 44 49)	SMS (2), Phone-based (1), VST (2)	“The flexibility [of the technology] is good. You can be texting a patient. They have a problem. You go and find that [specialist] in the clinic. You call and give the phone right to the health worker. They get immediate feedback!” (Phone-based)^17^
Convenience compared with alternative care models	20 (1718 22 28,35 37)	SMS (1), Phone-based (2), 99DOTS (1), Digital pillbox (3), VST (13)	“Coming every day to the hospital is definitely more difficult than learning to connect to a conference on whatever a laptop, tablet. I mean, it’s pretty flexible…” (VST)^43^
Improved quality of care	5 (17 21 37 38 42)	SMS (1), Phone-based (1), 99DOTS (1), VST (2)	
Ease of use			
DAT was easy to use	13 (20 25 26 32 33 35 36 43 47 48 54 59 87)	SMS (1), Digital pillbox (1), Ingestion sensors (1), VST (8), App-based (3)	
DAT provided flexibility and choice (eg, timing of observation, monitoring approach)	3 (27 44 46)	VST (3)	
Social influences			
Empowerment or enhanced autonomy	5 (22 38 59 88 90)	SMS (2), VST (1), App-based (2)	“Through blogging you can share thoughts without thinking of what others will think. It helps to write what happened and what my experience was…” (App-based)^88^
Lower or reduced stigma from DAT use	14 (1928 29 31,35 38 41 44 47)	SMS (2), Digital Pillbox (3), VST (9)	“(P)eople undermine and discriminate against you when you are on [HIV antiretroviral therapy], so the device is very helpful because nobody can see what is inside.” (Digital pillbox)^47^
Positive family involvement in care or social support	7 (4142 48 68 88,90)	SMS (1), Phone-based (2), 99DOTS (1), Digital pillbox (1), VST (1), App-based (1)	“[My son) taught me, ‘You have to take 2 tablets per day and follow the arrow mark from the starting point’…He gave me my medicines from the beginning [of therapy] and reminds me to take tablets…he also dials the toll-free numbers for me.” (99DOTS)^42^
Facilitating conditions			
Adequate DAT training by providers enabled engagement	2 (33 41)	Digital pillbox (1), VST (1)	
Provision of cellphone enabled DAT engagement	1 (48)	VST (1)	
Optimal DAT function or DAT problems easily resolved	4 (27 33 44 56)	SMS (1), VST (3)	
No or only minor challenges with network function	2 (33 44)	VST (2)	

App, application; DAT, digital adherence technology; SMS, short messaging service; TB, tuberculosis; UTAUT, Unified Theory of Acceptance and Use of TechnologyVSTvideo-supported therapy

Three meta-themes within perceived usefulness pertained to the impact of DATs on relationships between people with TB and their healthcare providers: facilitated communication with providers, improved relationship between the person with TB and providers, and fostered a feeling of being cared for. Of these, facilitated communication with providers was most frequently reported; people with TB valued having timely access to providers to ask questions about adverse effects, TB transmission risk and appointment scheduling.^17^,^28^

Three meta-themes within perceived usefulness pertained to perceptions of convenience and quality of care: convenience of care delivery, convenience compared with alternative care models and improved quality of care. People with TB most frequently reported improved convenience of care in relation to more restrictive care models, such as facility-based DOT, in which people must visit clinics multiple times a week for observation of dosing. In contrast, DATs minimised life disruptions by reducing clinic visits, providing flexibility in dosing timings and increasing treatment accessibility for people in rural areas or with demanding work schedules.^1718 22 28^,^45^

With regard to the UTAUT construct of ease of use, two meta-themes emerged: the DAT was easy to use and the DAT provided flexibility and choice. People valued flexibility in how and when they could report doses taken with DATs, for example when VST allowed for asynchronous options.^27 44 46^

With regard to the UTAUT construct of social influences, three meta-themes emerged: empowerment or enhanced autonomy, low or reduced stigma from DAT use and positive family involvement in care or social support. In several studies, people with TB experienced lower stigma due to perceptions of higher privacy and confidentiality of digital observation of dosing, usually in comparison to more restrictive in-person DOT models.^1928 29 31^,^35 38 41 44 47^

With regard to the UTAUT construct of facilitating conditions, four meta-themes emerged, suggesting that DAT engagement by people with TB could be supported by adequate training in the DAT by healthcare providers, provision of cellphones (for phone-based DATs), provision DATs that function optimally or for which problems are easily resolvable and settings with high-functioning cellular networks.

### Contextual factors limiting DAT reach among people with TB

Several meta-themes related to the UTAUT construct of perceived usefulness (table 3; findings for each reach meta-theme are in online supplemental appendix 2). Some people with TB felt DATs did not address their problems, because the technologies inadequately addressed critical challenges contributing to non-adherence, such as medication adverse effects, alcohol use or drug stockouts.^17 50 51^ Technology fatigue was also reported across multiple studies, as people with TB sometimes felt overwhelmed by frequent notifications and texting or calling requirements.^22 42 52 53^

**Table 3:** Meta-themes *limiting* reach (ie, DAT use by people with TB) with findings organised using the UTAUT

Emerging meta-themes divided by UTAUT construct	Number of studies reporting meta-theme (citations)	DATs for which finding was reported (Number of studies per DAT)	Representative quotation on the meta-theme
Perceived usefulness			
Greater access to information desired	2 (20 22)	SMS (1), App-based (1)	
DAT does not address patient problems	3 (17 50 51)	Phone-based (3)	
Technology fatigue	5 (22 42 52 53 66)	SMS (2), 99DOTS (3)	“My interest in calling has decreased compared to the initial phase…now I don’t [call after taking medication].” (99DOTS)^42^
DAT had physical adverse effects	1 (30)	Ingestion Sensor (1)	
Different or modified DAT modality desired	5 (20 22 37 61 66)	SMS (3), VST (1), App-based (1)	“[The system) was focused only on reminding…It would have been good if you also include other related messages, e.g. nutritional messages and others.” (SMS)^66^
Purpose of DAT monitoring is unclear	2 (42 53)	99DOTS (2)	
Negative impact on relationship with healthcare system	4 (34 42 53 63)	99DOTS (2), Digital pillbox (1), VST (1)	
Preference for in-person healthcare provider–patient communication	3 (18 22 59)	SMS (1), Phone-based (1), App-based (1)	
Concerns about surveillance	2 (41 47)	Digital pillbox (2)	
Ease of use			
Complex cellular accessibility challenges (at an individual level)	14 (1719 21,23 27 34 43 52)	SMS (6), Phone-based (1), 99DOTS (2), VST (5)	“I am staying in a hut, so I don’t have electricity in my home; we burn (wood) sticks to get light.” (99DOTS)^42^“My mother used to take [the cellphone] with her…for those days I couldn’t call… After taking medication I would tell my brother-in-law… But he ended up losing his SIM card.” (99DOTS)^42^“Sometimes due to [cellular] signal problems, although I was opening the box, these doses were not reported.” (Digital pillbox)^41^
Literacy and language barriers	10 (16,1820 42 49 53 57)	SMS (3), Phone-based (2), 99DOTS (3), App-based (2)	Healthcare provider observation on DAT engagement by people with TB: “(U)neducated illiterate…elderly grandmothers and grandfathers will be there…They will [say], ‘…(W)e don’t know how to operate the mobile phone…(W)e don’t have anyone; we don’t have any mobile [phone]’…In such situations we don’t know what to do.” (99DOTS)^53^
DAT too technically complex or physically difficult to use	10 (19 20 24 28 42 47 49 52 53 60)	SMS (2), 99DOTS (3), Digital Pillbox (2), VST (1), App-based (2)	“[A f]ew had difficulty in using those 10 digit numbers to call…(T)hey would have given some relatives the phone number. But the relative may not be there every time [when the patient takes medications]” (99DOTS)^53^
Size of DAT is prohibitive	4 (41 61 63 93)	Digital pillbox (4)	
Social influences			
High or increased stigma	17 (1718 20 22 26 28 35 38 41 42 44 48 53 57 60,62)	SMS (2), Phone-based (2), 99DOTS (2), Digital pillbox (3), VST (7), App-based (1)	“(I) tell [my friends] that I bought [the phone] myself…I don’t tell them that I got it from [the clinic], because my friends don’t know about my illness…it is a secret.” (Phone-based)^18^“I am very worried about my children coming to know [about the TB diagnosis]… so I am making calls while hiding from others.” (99DOTS)^42^
Desire for greater social contact via DAT with other people with TB	1 (20)	App-based (1)	
Concerns about family involvement in care	4 (28 41 53 55)	99DOTS (1), Digital pillbox (2), VST (1)	
Social events or holidays reduce DAT engagement	1 (92)	Digital pillbox (1)	
Socially desirable behaviour to please provider leads to inaccurate adherence data	1 (64)	Digital pillbox (1)	
Concerns about the high monetary value of the DAT	1 (61)	Digital pillbox (1)	
Facilitating conditions			
Suboptimal DAT function	12 (1821 24 28 37 41 42 44 53 63,65)	SMS (1), Phone-based (1), 99DOTS (2), Digital pillbox (4), VST (3), App-based (1)	“The first time I call, it gives a busy signal…after thinking that I dialed a wrong number.” (99DOTS)^42^
Complex cellular accessibility challenges (at a systemic level)	15 (1819 27 28 41,43 49 51 53 56 61 62 66 67)	SMS (5), Phone-based (3), 99DOTS (2), Digital Pillbox (2), VST (4)	“(L)ast week, I received all messages, but this week, I received only two messages. Our electric power and mobile network was not stable.” (Phone-based)^51^
Lack of adequate counselling on DAT	4 (41 42 52 53)	99DOTS (3), Digital Pillbox (1)	
Suboptimal culture or quality of general TB care leads to poor DAT implementation	3 (17 21 41)	SMS (1), Phone-based (1), Digital pillbox (1)	
Equity challenges	9 (17 41 47 52 54 56 57 68 69)	SMS (3), Phone-based (3), 99DOTS (2) Digital Pillbox (1)	“We are poor people. I have now started a job, before I was unemployed. So, if I have money, then I respond. When I don’t have [money] then I don’t send a response.” (SMS)^57^

App, application; DAT, digital adherence technology; SMS, short messaging service; TB, tuberculosis; UTAUT, Unified Theory of Acceptance and Use of Technology; VST, video-supported therapy

With regard to the UTAUT construct of ease of use, meta-themes had findings from several studies, suggesting that these barriers strongly limit DAT reach. People with TB reported numerous challenges to cellular accessibility at an individual level, including lack of cellphone access, shared cellphone use within the family, running out of credit, challenges refilling credit (ie, financial costs of DATs) and lack of a stable cellular or internet signal.^1719 21^,^23 27 34 43 52^ Literacy and language barriers were also diverse and included SMS texts sent in foreign languages, struggles comprehending technical language and reliance on family to interpret messages.^16^,^1820 42 49 53 57^

With regard to the UTAUT construct of social influences, the most frequently reported meta-themes were stigma and concerns about family involvement in care. Stigma often resulted from the increased visibility of pill-taking with DATs. For example, people with TB reported privacy concerns from making phone or video calls to report dose ingestion and that receiving a new phone or digital pillbox marked them as having TB or HIV.^1718 20 22 26 28 35 38 41 42 44 48 53 57 60^,^62^

With regard to the UTAUT construct of facilitating conditions, the most frequently reported meta-themes were suboptimal DAT function, cellular accessibility challenges at a systemic level and equity challenges. Suboptimal DAT function included problems such as cellphone calls not connecting (for 99DOTS)^42 53^; loud reminder alarms, malfunctioning reminder lights or discrepant reminder timings (for digital pillboxes)^28 41 63 64^; device battery failures and difficulties accessing a DAT App.^18 21 24 37 44 65^ Cellular accessibility challenges at a systemic level included inconsistent power supply and weak or inconsistent internet or cellular signals.^1819 27 28 41^,^43 49 51 53 56 61 62 66 67^ Equity challenges included differences in the ability of people with TB to access or engage with DATs by sex, age, educational status, income level and urban versus rural location.^17 42 47 52 54 56 57 68 69^

### Contextual factors promoting DAT adoption by healthcare providers

With regard to the UTAUT construct of perceived usefulness, meta-themes with the most findings promoting adoption were improved quality of care for people with TB, improved communication between providers and people with TB, better efficiency of care delivery and improved data quality (table 4; findings informing each adoption meta-theme are in online supplemental appendix 3). Healthcare providers valued DATs when they improved quality of care, through improved adherence monitoring, response to questions from people with TB or detection of medication adverse effects (reported as a separate meta-theme).^19 23 27 37 38 42 44 70^ Improved efficiency of care delivery due to DATs manifested as time savings and reduced transportation costs from not conducting in-person DOT and an ability to care of more people per provider, which led to reductions in staff, alleviation of clinic crowding or increased clinic capacity.^1923 27 29 38 40^,^42 44 59 71^

**Table 4:** Meta-themes *promoting* adoption (ie, DAT uptake by healthcare providers) with findings organised using the UTAUT

Emerging meta-themes divided by UTAUT construct	Number of studies reporting meta-theme (citations)	DATs for which finding was reported (number of studies per DAT)	Representative quotation on the meta-theme
Perceived usefulness			
Improved quality of care for people with TB	7 (19 23 37 38 42 44 70)	SMS (2), 99DOTS (1), Digital pillbox (1), VST (3)	“The health visitor and TB officers were coming to know who had missed doses in a span of one or two successive days. So, it became easy for the staff to contact those patients.” (99DOTS)^42^
Improved monitoring of medication adverse effects	3 (27 37 42)	99DOTS (1), VST (2)	
Improved communication between providers and people with TB	4 (19 21 42 44)	SMS (2), 99DOTS (1), VST (1)	
Improved relationship with people with TB	3 (24 42 88)	99DOTS (1), App-based (2)	
Increased job satisfaction	3(17 41 71)	Phone-based (1), Digital pillbox (2)	
Better efficiency of care delivery	13 (1923 27 29 38 40,42 44 59 71)	SMS (2), 99DOTS (1), Digital pillbox (2), VST (7), App-based (1)	“[VST] helps because a lot of the time we’re short [staffed and]…you don’t want your workers running around the streets all day.” (VST)^38^“I have more time now to check whether patients have taken their tablets…I am also able to concentrate on other tasks.” (Digital pillbox)^41^
Improved data quality	4 (21 42 59 71)	SMS (1), 99DOTS (1), Digital pillbox (1), App-based (1)	
Ease of use			
DAT is easy to learn and use	3 (42 44 71)	99DOTS (1), Digital pillbox (1), VST (1)	
Patient data easily accessible	3 (42 59 71)	99DOTS (1), Digital pillbox (1), App-based (1)	
Social influences			
Improved culture of care and communication among providers	4 (17 21 42 44)	SMS (1), Phone-based (1), 99DOTS (1), VST (1)	
Facilitating conditions			
Strong training, participation and care culture for providers	4 (17 37 44 71)	Phone-based (1), Digital pillbox (1), VST (2)	
DAT problems easily resolved	1 (27)	VST (1)	
Research evidence enhances implementation	1 (17)	Phone-based (1)	

App, application; DAT, digital adherence technology; SMS, short messaging service; TB, tuberculosis; UTAUT, Unified Theory of Acceptance and Use of TechnologyVSTvideo-supported therapy

With regard to the UTAUT construct of ease of use, meta-themes that emerged reflected provider appreciation of DATs that were easy to learn and use and that made data from people with TB easily accessible.^42 44 59 71^ With regard to the UTAUT construct of social influences, one meta-theme emerged, namely appreciation of DATs that improved the culture of care and communication among healthcare providers.^17 21 42 44^ With regard to the UTAUT construct of facilitating conditions, the meta-theme with the most findings pertained to situations in which the DAT was implemented in the context of strong training (eg, with provision of computer skills or professional development), participation and care culture for providers (eg, settings where person-centred care was emphasised).^17 37 44 71^

### Contextual factors limiting DAT adoption by healthcare providers

With regard to the UTAUT construct of perceived usefulness, meta-themes with the most findings related to limiting DAT adoption were: negative impact on workload or employment and suboptimal accuracy of adherence data (table 5; findings informing each adoption meta-theme are in online supplemental appendix 3). DAT interventions sometimes increased the work required of providers (eg, time spent training people in DAT use) or raised concerns about job losses due to more efficient care delivery.^17 42 53 59 70 73^ Suboptimal accuracy of adherence data referred to challenges identifying people who were non-adherent to medications, due to inaccuracies in DAT dosing histories.^1737 44 53 63^,^65^ This sometimes led to providers simply presuming that people not reporting doses were taking medications correctly.

**Table 5:** Meta-themes *limiting* adoption (ie, DAT use by healthcare providers) with findings organised using the UTAUT

Emerging meta-themes divided by UTAUT construct	Number of studies reporting meta-theme(citations)	DATs for which finding was reported (number of studies per DAT)	Representative quotation on the meta-theme
Perceived usefulness			
Negative impact on workload or employment concerns	6 (17 42 53 59 70 73)	Phone-based (1), 99DOTS (2), Digital pillbox (1), VST (1), App-based (1)	“[HIV] drug delivery itself is a big load; we need to give drugs, counsel and also follow-up…(A)dding (99DOTS) has become a burden for us…Because of shortage of manpower, we cannot have a single person constantly looking after TB/HIV patients.” (99DOTS)^53^
Suboptimal accuracy of adherence data	7 (1737 44 53 63,65)	Phone-based (1), 99DOTS (1), Digital pillbox (2), VST (3)	Description of a situation faced by a person with TB that led to suboptimal accuracy of dosing information, leading to concerns by healthcare providers:“Since I started going to work, I haven’t been using [the digital pillbox] that much because I cannot bring it along. It’s very cumbersome…I put [the medications] in my backpack.” (Digital pillbox)^63^“Not more than 50% [of patients give a missed call]. But they will take tablet(s).” (99DOTS)^53^
Reduced in-person patient–provider interactions negatively impact care	2 (59 73)	VST (1), App-based (1)	
Ease of use			
Challenges contacting patients	1 (18)	Phone-based (1)	
Social influences			
Decreased communication among HCPs	2 (52 53)	99DOTS (2)	
Facilitating conditions			
Suboptimal DAT function	7 (19 41 42 52 53 65 73)	SMS (1), 99DOTS (3), Digital pillbox (1), VST (2)	“It takes 72 hours for the [digital pillbox] dashboard to show that the patient has taken medications. This makes it difficult for us to monitor the patient’s drug intake…We cannot take action as promptly…” (Digital pillbox)^41^
Loss or destruction of the DAT	4 (22 37 64 91)	SMS (1), Digital pillbox (2), VST (1)	
Inadequate availability of technology	1 (71)	Digital pillbox (1)	
Complex cellular accessibility challenges (systemic level)	6 (17 44 52 53 71 73)	Phone-based (1), 99DOTS (2), Digital pillbox (1), VST (2)	
Insufficient training or technology skills for providers	5 (17 42 52 53 71)	Phone-based (1), 99DOTS (3), Digital pillbox (1)	“There were few trainings…there was no joint training for both [TB clinic] and [HIV clinic] staff.” (99DOTS)^53^
Insufficient staff for DAT implementation	3 (17 42 53)	Phone-based (1), 99DOTS (2)	
Poor management of DAT programme	3 (42 52 53)	99DOTS (3)	
Suboptimal culture of care	2 (17 53)	Phone-based (1), 99DOTS (1)	
Government barriers to implementation or sustainment	3 (17 59 73)	Phone-based (1), VST (1), App-based (1)	
Equity challenges	1 (73)	VST (1)	

App, application; DAT, digital adherence technology; SMS, short messaging service; TB, tuberculosis; UTAUT, Unified Theory of Acceptance and Use of Technology; VST, video-supported therapy

With regard to the UTAUT construct of ease of use, one meta-theme emerged, regarding challenges contacting people with TB.^18^ Similarly, for the UTAUT construct of social influences, one meta-theme emerged on decreased communication among healthcare providers, specifically describing challenges coordinating care between TB and HIV programmes in India after 99DOTS implementation.^52 53^

With regard to the UTAUT construct of facilitating conditions, the four themes with the most frequent findings were suboptimal DAT function, loss or destruction of DATs, complexity of cellular accessibility challenges at a systemic level and insufficient training or technology skills. Suboptimal DAT function referred to challenges such as inability to register more than one phone number per patient, inability to add information for treatment supporters or infrequent updating of digital dosing histories.^19 41 42 52 53 65 73^ Complex cellular accessibility challenges at a systemic level were a major barrier to adoption. Problems included failure of DAT digital records due to power shortages, weak network coverage at clinical sites or problems with video quality or receipt.^17 44 52 53 71 73^

## Discussion

In this paper, we identified numerous contextual factors promoting or limiting the implementation of TB DATs, specifically the reach of these technologies among people with TB and their adoption by healthcare providers. These diverse contextual factors provide insights into reasons for suboptimal engagement of people with TB and healthcare providers as identified in the companion scoping review of DAT implementation outcomes,^8^ while also partly explaining the mixed effectiveness of DATs for improving TB clinical outcomes and their variable accuracy for measuring adherence, especially in low-income and lower-middle-income country settings.^5 6^ By providing insights into what people with TB and healthcare providers value in TB care, this review may also inform strategies for tailoring DAT interventions to enhance future reach, adoption and effectiveness.

Complex cellular accessibility challenges were a major cross-cutting meta-theme limiting both reach and adoption. These challenges help explain the variability in adoption and serial drop-offs in engagement adversely affecting DAT reach—due to poor cellphone access, lack of initial uptake and lack of sustained use—that were quantified in the companion DAT implementation outcomes review.^8^ Most of these findings were reported from low-income and lower-middle-income country settings with less robust cellular network coverage, though these barriers were also experienced in upper-income and upper-middle-income country settings. Although optimism around the global expansion of cellular networks has driven use of DATs in TB care, both DAT implementation scoping reviews suggest that the realities of cellular accessibility are more complicated and may undermine DAT intervention programmes. Cellular network coverage may be particularly challenging for people with TB, as TB disproportionately affects individuals experiencing socioeconomic disadvantage.^74 75^

Among people with TB, other meta-themes on barriers to ease of use that limited reach—such as literacy and language barriers, technical complexity of some DATs and suboptimal DAT function—may have contributed to technology fatigue. TB-related stigma was an important meta-theme limiting reach because use of DATs to support adherence (eg, via audible or visual reminders) or report dose ingestion often made the act of pill-taking more conspicuous to family or coworkers, especially when compared with self-administered therapy without DAT monitoring. Inequitable access to DATs—by sex, age, education, income or urban versus rural location—was another major meta-theme limiting reach. Although a recent study suggests that some DAT-based interventions could help reduce inequity in TB treatment outcomes,^76^ findings of differential access to DATs in some settings also raise concern that these interventions could increase inequity in outcomes in the overall population of people with TB.

Meta-themes promoting DAT reach also provide general insights into what people value in their TB care. The meta-theme with the most robust findings related to perceptions of improved medication adherence behaviour, with several studies reporting that DAT interventions helped people form habits around pill-taking. This finding is perhaps not surprising, given that supporting medication adherence is the main premise of DAT interventions.^1^ However, people with TB also valued other benefits of DATs. For example, people with TB appreciated when DATs enhanced their ability to communicate with healthcare providers, provided enhanced access to TB information, improved monitoring of medication adverse effects and fostered a general sense of being ‘cared for’ by the health system. This last finding resonates with previous research on digital pillboxes among people with HIV in Uganda, in which real-time monitoring was experienced as ‘being seen’ by the health system, thereby motivating people to ‘take responsibility’ for medication adherence.^77^

Our findings suggest that perceptions of DATs by people with TB were strongly shaped by their perceptions of alternative models of TB care. For example, alternative care models sometimes include clinic-based DOT, in which people with TB visit clinics daily for observation of dosing by healthcare providers, which may affect employment and autonomy for people with TB.^78 79^ When this care model was the point of reference, people with TB tended to perceive DAT-based care models as resulting in greater convenience and lower stigma. Conversely, if self-administered therapy (ie, people taking medications on their own at home) was the point of reference, people tended to report more technology fatigue and increased stigma with DAT-based care models. Similarly, a recent systematic review found that the cost-effectiveness of TB DATs was highly influenced by the alternative care model to which the DAT-based model was being compared, with DAT-based models being more likely to be cost-effective when compared with more intensive in-person DOT.^80^ As such, findings from DAT studies—whether related to convenience, stigma or cost-effectiveness—are often as much a reflection of challenges or benefits of prior or alternative care models as they are reflections of the DAT interventions themselves.^81^

With regard to adoption, healthcare providers valued DATs when they improved the efficiency of care, by enabling fewer providers to manage more people with TB in a less time-intensive and resource-intensive manner. Healthcare providers also valued potential improvements in quality of care due to DAT interventions, including benefits related to better identification of medication non-adherence, improved communication with people with TB and enhanced identification of medication adverse effects. Importantly, one meta-theme indicated that some DAT interventions also improved communication among healthcare providers themselves (ie, within the care team) in a manner that may have improved the overall culture of care delivery.

A major meta-theme limiting adoption by healthcare providers was negative impacts of DAT-based care models on provider workloads (eg, due to new needs to counsel people with TB on the DAT) or employment (eg, fear of losing their jobs due to increased efficiencies in care delivery due to DATs). Healthcare providers were also sometimes frustrated due to the suboptimal accuracy of adherence data (ie, digital dosing histories) compiled by DATs, consistent with problems with or variability in, DAT accuracy reported in prior studies and a recent systematic review.^6 82 83^ Under-reporting of dose-taking, even when people with TB usually were taking their medications, resulted in providers having to regularly reach out to most people with TB to further assess their medication adherence.^17^ In some settings, provider outreach to people with TB dropped off, as providers began to assume that most people who were not reporting doses were probably taking their medications.^53^ This finding highlights the importance of evaluating the accuracy of DATs in real-world implementation, as inaccurate digital dosing data undermines the confidence of healthcare providers in these interventions.

Strengths of this review include use of a unique methodological approach that systematically synthesised qualitative and quantitative contextual data to identify diverse meta-themes informing the reach and adoption of DATs. A limitation of this scoping review is that we did not rate study quality. However, most systematic reviews of qualitative research do not eliminate studies based on quality; instead, qualitative systematic reviews tend to accentuate findings from higher-quality studies, as lower-quality studies tend to have less data-rich findings, which we also found to be the case in this review.^9 15^ Another limitation of this review is that relatively few studies reported findings on DAT adoption by healthcare providers. This limitation highlights a need for further research on DAT adoption, especially given highly variable DAT adoption by healthcare providers in some settings.^8 84^

## Conclusions

This scoping review has identified diverse contextual factors that may influence the reach of DATs among people with TB and adoption of these technologies by healthcare providers. These findings may inform selection of appropriate settings for DAT implementation, redesign of DATs to better address the needs of people with TB and healthcare providers, and implementation strategies to improve DAT uptake. A major finding of this review is that, despite increasing access to cellular networks globally, people with TB face diverse challenges to cellular accessibility that may undermine the effectiveness of DAT interventions, especially in low-income and middle-income country settings. As such, selection of settings with high cellular accessibility, or efforts to increase cellular accessibility (eg, provision of cellphones) may be critical to improve the reach and effectiveness of DAT interventions.

While people with TB value potential benefits of DATs for improving medication adherence, they also value other benefits such as enhanced communication with healthcare providers, ability to report medication adverse effects, and use of DAT platforms to gain important information about TB, all of which may foster a sense of being care for by the health system. Similarly, in addition to improvements in the efficiency of care delivery, healthcare providers also value the ability to communicate more easily with people with TB and with other providers, which may improve the overall culture of care. Future DAT-based interventions should move beyond solely focusing on digital observation of dosing and measurement of adherence, by leveraging DATs to facilitate human interactions and address structural barriers to care.^3 85^ Focusing on what people with TB and healthcare providers value may enhance the future implementation, effectiveness and public health impact of these technologies.

## supplementary material

10.1136/bmjgh-2024-016608 online supplemental file 1

10.1136/bmjgh-2024-016608 online supplemental file 2

10.1136/bmjgh-2024-016608 online supplemental file 3

## Data Availability

All data relevant to the study are included in the article or uploaded as supplementary information.
