# Supplementary material for: Contextual factors influencing implementation of tuberculosis digital adherence technologies: a scoping review guided by the RE-AIM framework
Source: BMJ Glob Health. 2025 Feb 13;10(2):e016608. doi: 10.1136/bmjgh-2024-016608 (PMC11831270; doi:10.1136/bmjgh-2024-016608)
Supplement: online supplemental file 1 [file bmjgh-10-2-s001.pdf]

Online supplementary appendix 1
PRISMA scoping reviews checklist and search strategy

Supplement to:

Contextual factors impacting implementation of tuberculosis digital adherence technologies: a scoping review using the RE-AIM framework

S. Bahukudumbi, C. Chilala, B. Patel, N. Foster, M.S. Mohamed, M. Zary, C. Kafie, K. Schwartzman, K. Fielding, R. Subbaraman

Correspondence:
Ramnath Subbaraman, MD, MSc, FACP, FIDSA
Tufts University School of Medicine
Department of Public Health and Community Medicine
136 Harrison Ave., MV237
Boston, MA 02130, USA
Email: ramnath.subbaraman@tufts.edu

Table of Contents

Preferred Reporting Items for Systematic reviews and Meta-Analyses extension (PRISMA) abstract checklist 3

Preferred Reporting Items for Systematic reviews and Meta-Analyses extension for Scoping Reviews (PRISMA-ScR) checklist.. 5

Search strategy for the scoping review 8

Preferred Reporting Items for Systematic reviews and Meta-Analyses extension (PRISMA) abstract checklist

Section and Topic	Item #	Checklist item	Reported (Yes/No)
**TITLE**			
Title	1	Identify the report as a scoping review.	Yes
**BACKGROUND**			
Objectives	2	Provide an explicit statement of the main objective(s) or question(s) the review addresses.	Yes, second sentence of Introduction of Abstract
**METHODS**			
Eligibility criteria	3	Specify the inclusion and exclusion criteria for the review.	Yes, Methods section of Abstract, first sentence
Information sources	4	Specify the information sources (e.g. databases, registers) used to identify studies and the date when each was last searched.	Yes, Methods section of the Abstract, first sentence (8 databases were noted to have been searched, with details in the main manuscript)
Risk of bias	5	Specify the methods used to assess risk of bias in the included studies.	Not applicable for a scoping review
Synthesis of results	6	Specify the methods used to present and synthesise results.	Yes, Methods section of the Abstract, second and third sentences
**RESULTS**			

Section and Topic	Item #	Checklist item	Reported (Yes/No)
Included studies	7	Give the total number of included studies and participants and summarise relevant characteristics of studies.	Yes, Results section of Abstract, first sentence
Synthesis of results	8	Present results for main outcomes, preferably indicating the number of included studies and participants for each. If meta-analysis was done, report the summary estimate and confidence/credible interval. If comparing groups, indicate the direction of the effect (i.e. which group is favoured).	Yes, Results section of the Abstract, second, third, fourth, or fifth sentences
**DISCUSSION**			
Limitations of evidence	9	Provide a brief summary of the limitations of the evidence included in the review (e.g. study risk of bias, inconsistency and imprecision).	Yes, Results section of the Abstract, last sentence
Interpretation	10	Provide a general interpretation of the results and important implications.	Yes, Conclusion section of the Abstract
**OTHER**			
Funding	11	Specify the primary source of funding for the review.	Yes, this has been reported separately in the journal online system
Registration	12	Provide the register name and registration number.	Yes, this is reported later in the main manuscript

From: Page MJ, McKenzie JE, Bossuyt PM, Boutron I, Hoffmann TC, Mulrow CD, et al. The PRISMA 2020 statement: an updated guideline for reporting systematic reviews. BMJ 2021;372:n71. doi: 10.1136/bmj.n71

For more information, visit: http://www.prisma-statement.org/

Preferred Reporting Items for Systematic reviews and Meta-Analyses extension for Scoping Reviews (PRISMA-ScR) checklist

SECTION	ITEM	PRISMA-ScR CHECKLIST ITEM	REPORTED ON PAGE #
**TITLE**			
Title	1	Identify the report as a scoping review.	Reported on the title page
**ABSTRACT**			
Structured summary	2	Provide a structured summary that includes (as applicable): background, objectives, eligibility criteria, sources of evidence, charting methods, results, and conclusions that relate to the review questions and objectives.	Reported in the Abstract. The PRISMA Abstract checklist has also been completed.
**INTRODUCTION**			
Rationale	3	Describe the rationale for the review in the context of what is already known. Explain why the review questions/objectives lend themselves to a scoping review approach.	Introduction, paragraphs 2, 3, and 4
Objectives	4	Provide an explicit statement of the questions and objectives being addressed with reference to their key elements (e.g., population or participants, concepts, and context) or other relevant key elements used to conceptualize the review questions and/or objectives.	Introduction, paragraph 5
**METHODS**			
Protocol and registration	5	Indicate whether a review protocol exists; state if and where it can be accessed (e.g., a Web address); and if available, provide registration information, including the registration number.	Methods section, subsection on "PICOS framework and scoping review design," paragraph 1
Eligibility criteria	6	Specify characteristics of the sources of evidence used as eligibility criteria (e.g., years considered, language, and publication status), and provide a rationale.	Methods section, subsection on "Inclusion and exclusion criteria" and "PICOS framework and scoping review design," paragraph 2
Information sources*	7	Describe all information sources in the search (e.g., databases with dates of coverage and contact with authors to identify additional sources), as well as the date the most recent search was executed.	Methods section, subsection on "Search strategy"
Search	8	Present the full electronic search strategy for at least 1 database, including any limits used, such that it could be repeated.	Online supplementary appendix 1
Selection of sources of evidence†	9	State the process for selecting sources of evidence (i.e., screening and eligibility) included in the scoping review.	Methods section, subsection on "Screening strategy and study selection"

SECTION	ITEM	PRISMA-ScR CHECKLIST ITEM	REPORTED ON PAGE #
Data charting process‡	10	Describe the methods of charting data from the included sources of evidence (e.g., calibrated forms or forms that have been tested by the team before their use, and whether data charting was done independently or in duplicate) and any processes for obtaining and confirming data from investigators.	Methods section, subsection on “Data extraction,” first paragraph
Data items	11	List and define all variables for which data were sought and any assumptions and simplifications made.	Methods section, subsection on “Data extraction” fourth and fifth sentences
Critical appraisal of individual sources of evidence§	12	If done, provide a rationale for conducting a critical appraisal of included sources of evidence; describe the methods used and how this information was used in any data synthesis (if appropriate).	Methods section, subsection on “Data analysis: synthesis of meta-themes and reporting using the UTAUT,” paragraph 2, sentences 3 and 4, which provide a rationale for not evaluating study quality
Synthesis of results	13	Describe the methods of handling and summarizing the data that were charted.	Methods section, subsection on “Data analysis: synthesis of meta-themes and reporting using the UTAUT” (entire subsection)
**RESULTS**			
Selection of sources of evidence	14	Give numbers of sources of evidence screened, assessed for eligibility, and included in the review, with reasons for exclusions at each stage, ideally using a flow diagram.	Results section, subsection on “Characteristics of the included studies,” paragraph 1, Figure 1 (PRISMA flowchart), and Table 1
Characteristics of sources of evidence	15	For each source of evidence, present characteristics for which data were charted and provide the citations.	Results section, subsection on “Characteristics of the included studies,” paragraph 1 and Table 1
Critical appraisal within sources of evidence	16	If done, present data on critical appraisal of included sources of evidence (see item 12).	Results section, subsection on “Characteristics of the included studies,” paragraph 1 and Table 1
Results of individual sources of evidence	17	For each included source of evidence, present the relevant data that were charted that relate to the review questions and objectives.	Online supplementary appendices 2 and 3, which provide details on findings from the sources of evidence that inform meta-themes for “reach” and “adoption”
Synthesis of results	18	Summarize and/or present the charting results as they relate to the review questions and objectives.	Tables 2, 3, 4, and 5, which present meta-themes informing “reach” and “adoption”
**DISCUSSION**			
Summary of evidence	19	Summarize the main results (including an overview of concepts, themes, and types of evidence available), link to the review questions and objectives, and consider the relevance to key groups.	Discussion section, paragraphs 1 to 7
Limitations	20	Discuss the limitations of the scoping review process.	Discussion section, paragraph 8

SECTION	ITEM	PRISMA-ScR CHECKLIST ITEM	REPORTED ON PAGE #
Conclusions	21	Provide a general interpretation of the results with respect to the review questions and objectives, as well as potential implications and/or next steps.	Conclusions section, paragraphs 1 and 2
**FUNDING**			
Funding	22	Describe sources of funding for the included sources of evidence, as well as sources of funding for the scoping review. Describe the role of the funders of the scoping review.	Funding section

JBI = Joanna Briggs Institute; PRISMA-ScR = Preferred Reporting Items for Systematic reviews and Meta-Analyses extension for Scoping Reviews.
* Where *sources of evidence* (see second footnote) are compiled from, such as bibliographic databases, social media platforms, and Web sites.
† A more inclusive/heterogeneous term used to account for the different types of evidence or data sources (e.g., quantitative and/or qualitative research, expert opinion, and policy documents) that may be eligible in a scoping review as opposed to only studies. This is not to be confused with *information sources* (see first footnote).
‡ The frameworks by Arksey and O'Malley (6) and Levac and colleagues (7) and the JBI guidance (4, 5) refer to the process of data extraction in a scoping review as data charting.
§ The process of systematically examining research evidence to assess its validity, results, and relevance before using it to inform a decision. This term is used for items 12 and 19 instead of "risk of bias" (which is more applicable to systematic reviews of interventions) to include and acknowledge the various sources of evidence that may be used in a scoping review (e.g., quantitative and/or qualitative research, expert opinion, and policy document).

From: Tricco AC, Lillie E, Zarin W, O'Brien KK, Colquhoun H, Levac D, et al. PRISMA Extension for Scoping Reviews (PRISMAScR): Checklist and Explanation. Ann Intern Med. 2018;169:467–473. doi: 10.7326/M18-0850.

Search strategy for the scoping review

MEDLINE (Ovid)

Ovid MEDLINE(R) ALL <1946 to April 20, 2023>

1 exp tuberculosis/ or mycobacterium tuberculosis/ 228085

2 Antitubercular Agents/ 41252

3 (Tuberculosis or Kochs disease or Phthisis or TB or MTB or MDRTB or XDRTB or DRTB or LTBI or directly observed treatment short course).mp. 300942

4 1 or 2 or 3 [TB CONCEPT] 306368

5 ((antitubercular agents/ or directly observed therapy/ or medication adherence/ or patient compliance/ or "treatment adherence and compliance"/) and technology/) or mobile applications/ or internet/ or cell phone/ or smartphone/ or text messaging/ or computer, handheld/ or telemedicine/ or therapy, computer-assisted/ or medical informatics applications/ 148180

6 (((digital* or electronic* or mobile or wireless* or virtual*) adj2 (adherence or medication monitor* or medication package* or observ*)) or digital technolog* or technology-based or Digital health or eHealth or e health or mhealth or m health or SMS or reminder* or short messag* service* or text messag* or MMS or multimedia messag* or MEMS or (monitor* adj2 (electronic* or sensor* or device*)) or Webcam* or web cam* or smartphone* or smart phone* or web based or health IT or health ICT or ((cell* or mobile) adj1 (device* or health or phone* or technolog*)) or video* or cellphone* or feature phone* or vdot or vmalt or tele* or dat or wot or 99dots or 99 dots or monitoring system* or ingestible sensor* or merm or artificial intelligence or ai or vot or ((digital or smart) adj (pill box* or pillbox*)) or VST).mp. 705541

7 5 or 6 [DAT CONCEPT] 772972

8 4 and 7 [TB CONCEPT AND DAT CONCEPT] 3221

9 limit 8 to yr="2000 -Current" 2793

10 ("20220414" or "20220415" or "20220416" or "20220417" or "20220418" or "20220419" or 2022042* or 2022043* or 202205* or 202206* or 202207* or 202208* or 202209* or 20221* or 2023*).dt,ez,da. 1903196

11 9 and 10 354

https://proxy.library.mcgill.ca/login?url=https://ovidsp.ovid.com/ovidweb.cgi?T=JS&NEWS=N&PAGE=main&SHAREDSEARCHID=3fnvVdcJME85FE7alzUoGwgVxZWFQGclstIdKYE9CMQFMhddHUd1jxgKmo1hQDBKW

Embase (Ovid)

Embase <1996 to 2023 Week 15>

1 exp tuberculosis/ or mycobacterium tuberculosis/ 204337

2 tuberculostatic agent/ 30987

3 (Tuberculosis or Kochs disease or Phthisis or TB or MTB or MDRTB or XDRTB or DRTB or LTBI or directly observed treatment short course).mp. 246187

4 1 or 2 or 3 [TB CONCEPT] 257637

5 ((tuberculostatic agent/ or directly observed therapy/ or medication compliance/ or patient compliance/) and technology/) or communication technology/ or exp mobile application/ or internet/ or web-based intervention/ or exp mobile phone/ or text messaging/ or personal digital assistant/ or telemedicine/ or exp teleconsultation/ or telemonitoring/ or video consultation/ or computer-assisted therapy/ or computer-assisted drug therapy/ or medical informatics/ 261570

6 (((digital* or electronic* or mobile or wireless* or virtual*) adj2 (adherence or medication monitor* or medication package* or observ*)) or digital technolog* or technology-based or Digital health or eHealth or e health or mhealth or m health or SMS or reminder* or short messag* service* or text messag* or MMS or multimedia messag* or MEMS or (monitor* adj2 (electronic* or sensor* or device*)) or Webcam* or web cam* or smartphone* or smart phone* or web based or health IT or health ICT or ((cell* or mobile) adj1 (device* or health or phone* or technolog*)) or video* or cellphone* or feature phone* or vdot or vmalt or tele* or dat or wot or 99dots or 99 dots or monitoring system* or ingestible sensor* or merm or artificial intelligence or ai or vot or ((digital or smart) adj (pill box* or pillbox*)) or VST).mp. 870210

7 5 or 6 [DAT CONCEPT] 984663

8 4 and 7 [TB CONCEPT AND DAT CONCEPT] 5167

9 limit 8 to yr="2000 -Current" 5020

10 limit 9 to dc=20220414-20230419 661

https://proxy.library.mcgill.ca/login?url=https://ovidsp.ovid.com/ovidweb.cgi?T=JS&NEWS=N&PAGE=main&SHAREDSEARCHID=7KKR39ofVynipL3CX0ToO3GWb5GrslsDhbjFanUYdIRYp9ugDOcxhfduODOtnrlX1

CINAHL (EBSCOhost)

#	Query	Limiters/Expanders	Last Run Via	Results
S11	S9 AND S10	Expanders - Apply equivalent subjects Search modes - Boolean/Phrase	Interface - EBSCOhost Research Databases Search Screen - Advanced Search Database - CINAHL Plus with Full Text	146
S10	EM 20220414- OR ZD "in process"	Expanders - Apply equivalent subjects Search modes - Boolean/Phrase	Interface - EBSCOhost Research Databases Search Screen - Advanced Search Database - CINAHL Plus with Full Text	1,147,669
S9	S4 AND S7	Limiters - Published Date: 20000101-20231231 Expanders - Apply equivalent subjects Search modes - Boolean/Phrase	Interface - EBSCOhost Research Databases Search Screen - Advanced Search Database - CINAHL Plus with Full Text	857
S8	S4 AND S7	Expanders - Apply equivalent subjects	Interface - EBSCOhost Research	916

		Search modes - Boolean/Phrase	Databases Search Screen - Advanced Search Database - CINAHL Plus with Full Text	
S7	S5 OR S6	Expanders - Apply equivalent subjects Search modes - Boolean/Phrase	Interface - EBSCOhost Research Databases Search Screen - Advanced Search Database - CINAHL Plus with Full Text	347,115
S6	(((digital* OR electronic* OR mobile OR wireless* OR virtual*) N2 (adherence OR "medication monitor*" OR "medication package*" OR observ*)) OR "digital technolog*" OR technology-based OR "Digital health" OR eHealth OR "e health" OR mhealth OR "m health" OR SMS OR reminder* OR "short messag* service*" OR "text messag*" OR MMS OR "multimedia messag*" OR MEMS OR (monitor* N2 (electronic* OR sensor* OR device*)) OR Webcam* OR "web cam*" OR smartphone* OR "smart phone*" OR "web based" OR "health IT" OR "health ICT" OR ((cell* OR mobile) N1 (device* OR health OR phone* OR technolog*)) OR video* OR cellphone* OR "feature phone*" OR vdot OR vmalt OR tele* OR dat OR wot OR 99dots OR S99 dots OR	Expanders - Apply equivalent subjects Search modes - Boolean/Phrase	Interface - EBSCOhost Research Databases Search Screen - Advanced Search Database - CINAHL Plus with Full Text	278,594

	"monitoring system*" OR "ingestible sensor*" OR merm OR "artificial intelligence" OR ai OR vot OR ((digital OR smart) W1 ("pill box*" OR pillbox*)) OR VST)			
S5	((MH "Antitubercular Agents") OR (MH "Directly Observed Therapy") OR (MH "Medication Compliance") OR (MH "Patient Compliance")) AND (MH "Technology")) OR (MH "Wireless Communications") OR (MH "Mobile Applications") OR (MH "Internet") OR (MH "Internet-Based Intervention") OR (MH "Cellular Phone+") OR (MH "Text Messaging+") OR (MH "Instant Messaging") OR (MH "Interactive Voice Response Systems") OR (MH "Videoconferencing+") OR (MH "Computers, Hand-Held+") OR (MH "Telehealth+") OR (MH "Digital Technology") OR (MH "Therapy, Computer Assisted") OR (MH "Drug Therapy, Computer Assisted") OR (MH "Medical Informatics")	Expanders - Apply equivalent subjects Search modes - Boolean/Phrase	Interface - EBSCOhost Research Databases Search Screen - Advanced Search Database - CINAHL Plus with Full Text	133,190
S4	S1 OR S2 OR S3	Expanders - Apply equivalent subjects Search modes - Boolean/Phrase	Interface - EBSCOhost Research Databases Search Screen - Advanced Search Database - CINAHL Plus with Full Text	39,412
S3	(Tuberculosis OR "Kochs disease" OR Phthisis OR TB OR MTB OR MDRTB OR XDRTB OR DRTB OR	Expanders - Apply equivalent subjects	Interface - EBSCOhost Research Databases	39,091

	LTBI OR "directly observed treatment short course")	Search modes - Boolean/Phrase	Search Screen - Advanced Search Database - CINAHL Plus with Full Text	
S2	(MH "Antitubercular Agents")	Expanders - Apply equivalent subjects Search modes - Boolean/Phrase	Interface - EBSCOhost Research Databases Search Screen - Advanced Search Database - CINAHL Plus with Full Text	4,883
S1	(MH "Tuberculosis+") OR (MH "Mycobacterium Tuberculosis")	Expanders - Apply equivalent subjects Search modes - Boolean/Phrase	Interface - EBSCOhost Research Databases Search Screen - Advanced Search Database - CINAHL Plus with Full Text	26,642

CENTRAL (Cochrane Library/Wiley)

Search Name:

Date Run: 19/04/2023 19:42:11

Comment:

ID Search Hits

#1 (Tuberculosis or "Kochs disease" or Phthisis or TB or MTB or MDRTB or XDRTB or DRTB or LTBI or "directly observed treatment short course"):ti,ab,kw 9141

#2 (((digital* or electronic* or mobile or wireless* or virtual*) NEAR/2 (adherence or medication NEXT monitor* or medication NEXT package* or observ*)) or digital NEXT technolog* or technology-based or "Digital health" or eHealth or "e health" or mhealth or "m health" or SMS or reminder* or short NEXT messag* NEXT service* or text NEXT messag* or MMS or multimedia NEXT messag* or MEMS or (monitor* NEAR/2 (electronic* or sensor* or device*)) or Webcam* or web NEXT cam* or smartphone* or smart NEXT phone* or "web based" or "health IT" or "health ICT" or ((cell* or mobile) NEXT (device* or health or phone* or technolog*)) or video* or cellphone* or feature NEXT phone* or vdot or vmalt or tele* or dat or wot or 99dots or 99 NEXT dots or monitoring NEXT system* or ingestible NEXT sensor* or merm or artificial NEXT intelligence or ai or vot or ((digital or smart) NEXT (pill box* or pillbox*)) or VST):ti,ab,kw 359210

#3 #1 AND #2 with Publication Year from 2000 to 2023, in Trials 2118

Date added to CENTRAL trials database

Custom Range: 14/04/2022 to 19/04/2023

196 of 2118 records

WOS.SCI,WOS.ISTP,WOS.ESCI (Web of Science Core Collection)

Web of Science Search Strategy (v0.1)

Search: TS=(Tuberculosis OR "Kochs disease" OR Phthisis OR TB OR MTB OR MDRTB OR XDRTB OR DRTB OR LTBI OR "directly observed treatment short course")

AND

TS=(((digital* OR electronic* OR mobile OR wireless* OR virtual*) NEAR/2 (adherence OR "medication monitor*" OR "medication package*" OR observ*)) OR "digital technolog*" OR technology-based OR "Digital health" OR eHealth OR "e health" OR mhealth OR "m health" OR SMS OR reminder* OR "short messag* service*" OR "text messag*" OR MMS OR "multimedia messag*" OR MEMS OR (monitor* NEAR/2 (electronic* OR sensor* OR device*)) OR Webcam* OR "web cam*" OR smartphone* OR "smart phone*" OR "web based" OR "health IT" OR "health ICT" OR ((cell* OR mobile) NEAR/1 (device* OR health OR phone* OR technolog*)) OR video* OR cellphone* OR "feature phone*" OR vdot OR vmalt OR tele* OR dat OR wot OR 99dots OR "99 dots" OR "monitoring system*" OR "ingestible sensor*" OR merm OR "artificial intelligence" OR ai OR vot OR ((digital OR smart) NEAR/0 ("pill box*" OR pillbox*)) OR VST)

Editions: WOS.SCI,WOS.ISTP,WOS.ESCI

Timespan: 2000-01-01 to 2023-04-19

Date Run: Fri Apr 21 2023 14:19:23 GMT-0400 (Eastern Daylight Time)

Results: 2863

Database: Web of Science Core Collection

Entitlements:

- WOS.IC: 1993 to 2023

- WOS.CCR: 1985 to 2023

- WOS.SCI: 1900 to 2023

- WOS.AHCI: 1975 to 2023

- WOS.BHCI: 2005 to 2023

- WOS.BSCI: 2005 to 2023

- WOS.ESCI: 2005 to 2023

- WOS.ISTP: 1990 to 2023

- WOS.SSCI: 1900 to 2023

- WOS.ISSHP: 1990 to 2023

Searches:

Search:

#1

Timespan: 2022-04-14 to 2023-04-19

Date Run: Fri Apr 21 2023 14:20:33 GMT-0400 (Eastern Daylight Time)

Results: 329

MedRxiv and other preprints via Europe PMC

(TITLE:Tuberculosis OR TITLE:"Kochs disease" OR TITLE:Phthisis OR TITLE:TB OR TITLE:MTB OR TITLE:MDRTB OR TITLE:XDRTB OR TITLE:DRTB OR TITLE:LTBI OR TITLE:"directly observed treatment short course" OR TITLE:"antituberculosis" OR TITLE:"antituberculous") AND (Adher* OR "directly observed" OR Digital OR electronic OR

internet OR mobile OR wireless OR virtual OR TITLE:technology OR "technology based" OR tele* OR ehealth OR "e health" OR mhealth OR "m health" OR SMS OR reminder* OR messaging OR message* OR MEMS OR web OR webcam* OR smartphone* OR "health IT" OR "health ICT" OR video* OR cellphone* OR phone* OR vdot OR vmalt OR dat OR wot OR 99dots OR "99 dots" OR "monitoring system" OR "monitoring systems" OR "ingestible sensor" OR "ingestible sensors" OR merm OR "artificial intelligence" OR AI OR VOT OR "smart pillbox" OR "smart pill box" OR VST OR "computer assisted") AND (SRC:PPR) AND CREATION_DATE:[2022-04-19 TO 2023-04-19]

Clinicaltrials.gov basic search

Translated and exported to EndNote/RIS and documented

384 records on April 25, 2023

384 Studies found for: **Adherence OR directly observed OR computer OR Digital OR electronic OR internet OR mobile OR virtual OR technology OR video OR mhealth OR artificial intelligence OR AI OR cellphones OR SMS OR reminders OR monitoring OR MEMS OR DAT OR sensors | Tuberculosis OR Kochs disease OR Phthisis OR TB OR MTB OR MDRTB OR XDRTB OR DRTB OR LTBI OR directly observed treatment short course**
